# Direct and indirect effects of CYTOR lncRNA regulate HIV gene expression

**DOI:** 10.1371/journal.ppat.1012172

**Published:** 2024-04-25

**Authors:** Alona Kuzmina, Lopamudra Sadhu, Md Hasanuzzaman, Koh Fujinaga, Jacob C. Schwartz, Oliver T. Fackler, Ran Taube

**Affiliations:** 1 The Shraga Segal Department of Microbiology Immunology and Genetics, Faculty of Health Sciences, Ben-Gurion University of the Negev, Israel; 2 Department of Infectious Diseases, Heidelberg University, Medical Faculty Heidelberg, Integrative Virology, Center for Integrative Infectious Disease Research (CIID), Heidelberg, Germany; 3 Department of Medicine, University of California, San Francisco, San Francisco, California, United States of America; 4 Department of Pharmacology, University of Arizona College of Medicine, Tucson, Arizona, United States of America; 5 German Center for Infection Research, DZIF, Partner Site Heidelberg, Heidelberg. Germany; Boston College, UNITED STATES

## Abstract

The implementation of antiretroviral therapy (ART) has effectively restricted the transmission of Human Immunodeficiency Virus (HIV) and improved overall clinical outcomes. However, a complete cure for HIV remains out of reach, as the virus persists in a stable pool of infected cell reservoir that is resistant to therapy and thus a main barrier towards complete elimination of viral infection. While the mechanisms by which host proteins govern viral gene expression and latency are well-studied, the emerging regulatory functions of non-coding RNAs (ncRNA) in the context of T cell activation, HIV gene expression and viral latency have not yet been thoroughly explored.

Here, we report the identification of the Cytoskeleton Regulator (CYTOR) long non-coding RNA (lncRNA) as an activator of HIV gene expression that is upregulated following T cell stimulation. Functional studies show that CYTOR suppresses viral latency by directly binding to the HIV promoter and associating with the cellular positive transcription elongation factor (P-TEFb) to activate viral gene expression. CYTOR also plays a global role in regulating cellular gene expression, including those involved in controlling actin dynamics. Depletion of CYTOR expression reduces cytoplasmic actin polymerization in response to T cell activation. In addition, treating HIV-infected cells with pharmacological inhibitors of actin polymerization reduces HIV gene expression. We conclude that both direct and indirect effects of CYTOR regulate HIV gene expression.

## Author summary

A complete cure against HIV infection remains elusive, as the virus persists in stable cell reservoirs that are resistant to antiretroviral therapy. While the mechanisms by which host proteins govern viral gene expression and latency are extensively studied, the emerging regulatory roles of non-coding RNAs (ncRNA) in the context of T cell activation, HIV gene expression, and viral latency are less understood. We report the identification of novel ncRNAs that potentially play a role in regulating HIV gene expression in response to T-cell activation. Our work demonstrates that the Cytoskeleton Regulator (CYTOR) long non-coding RNA (lncRNA) exhibits a profound shift in RNA levels upon T-cell stimulation. Functional studies further reveal that CYTOR enhances HIV gene expression and prevents latency establishment following T cell stimulation. CYTOR directly binds to the HIV promoter and associates with the cellular positive transcription machinery to enhance viral gene expression. CYTOR also plays a global role in regulating cellular gene expression, including genes that are involved in the regulation of actin dynamics. Depletion of CYTOR reduces cytoplasmic actin polymerization in response to T cell stimulation. Increasing CYTOR levels did not alter the actin morphology formed in response to TCR activation. Concurrently, pharmacological inhibition of actin polymerization induced by T-cell activation reduces HIV gene expression.

## Introduction

The introduction of antiretroviral therapy (ART) has successfully limited the spread of Human Immunodeficiency Virus (HIV) and improved patient clinical outcomes. However, a complete cure for HIV infection remains out of reach, as the transcriptionally silent but replication-competent provirus that is integrated into the host genome persists in long-lived cellular reservoirs, which are comprised of memory-resting CD4^+^ T cells, as well as cells of myeloid lineages [1,2]. These reservoirs are highly stable and are resistant to both ART and the effects of the host immune surveillance, thus posing a significant obstacle to eradicating the HIV reservoirs. Consequently, in most people living with HIV, interrupting ART leads to rapid viral load rebound, usually within weeks after treatment cessation [3–6]. As T cell stimulation triggers activation of proviral transcription, one strategy that has been proposed to eliminate the HIV reservoirs is a “Shock-and-Kill” approach, which utilizes latency-reversing agents (LRAs) to first activate dormant HIV-infected T cells and facilitate cell death by viral cytopathic effects or immune-mediated killing. This step is done in the presence of ART, so there are no further rounds of HIV replication. [7–9]. Alternatively, a “Block and Lock” approach frees infected individuals from ART by silencing HIV transcription and inducing a deep state of latency. Nevertheless, despite promising therapeutic options, these strategies and others have regretfully failed to achieve significant clinical efficacy. These failures highlight our lack of knowledge of the molecular mechanisms that govern latency establishment and reversal and the need for alternative therapies capable of eliminating the viral reservoirs [10–15].

Epigenetic constraints that suppress proviral gene transcription are essential for establishing HIV latency [16,17]. Low levels of basal and elongating transcription factors in the infected T cell, together with the absence of the viral trans-activator of transcription (Tat), ensure that proviral transcription remains below detectable thresholds [18,19]. Within the infected T cells, gene transcription of the integrated provirus and the host genome are synchronized [20,21]. Both display key steps of gene transcription, which include initiation, promoter arrest, and elongation. HIV-Tat orchestrates transcription elongation of the provirus by binding to TAR RNA and recruiting P-TEFb and Super Elongation Complex (SEC) to the viral promoter [22–26]. However, despite extensive efforts to elucidate the mechanisms of metazoan transcriptional control and its role in the regulation of HIV gene transcription, the knowledge of how HIV latency is established is still incomplete [27].

Long non-coding RNAs (lncRNAs) are transcripts with longer than 200 nucleotides that lack protein-coding capacity. To date, over 200,000 cell type-specific lncRNAs have been identified and display critical regulatory functions of many processes within cells [28–31]. However, the functions of most of these transcripts remain poorly understood. In the context of HIV, roles for several cellular lncRNAs have been documented [32–40]. Moreover, significant gaps still remain in our knowledge about the mechanistic roles that lncRNAs play in CD4 T cell activation and HIV latency.

In this study, we monitored changes in gene expression in an HIV-infected Jurkat-derived T cell line (J-Lat 6.3) upon response to T cell stimulation with Phorbol 12-myristate 13-acetate—PMA/Ionomycin (P/I). We documented RNA expression in stimulated J-Lat 6.3 cells that carry either active or cells latent HIV, and among identified ncRNA, Cytoskeleton Regulator RNA (CYTOR) exhibited a profound change in expression in cells that expressed active HIV following T cell stimulation. CYTOR directly binds the HIV promoter and activates viral gene transcription and latency reversal by recruiting P-TEFb to the viral promoter. CYTOR also exerts its effects indirectly by controlling global gene expression along with actin dynamic pathways, thereby affecting T cell activation and HIV infection.

## Results

### Identification of lncRNAs that are differentially expressed following T-cell stimulation in T cells that carry active or latent HIV

**Fig 1:**
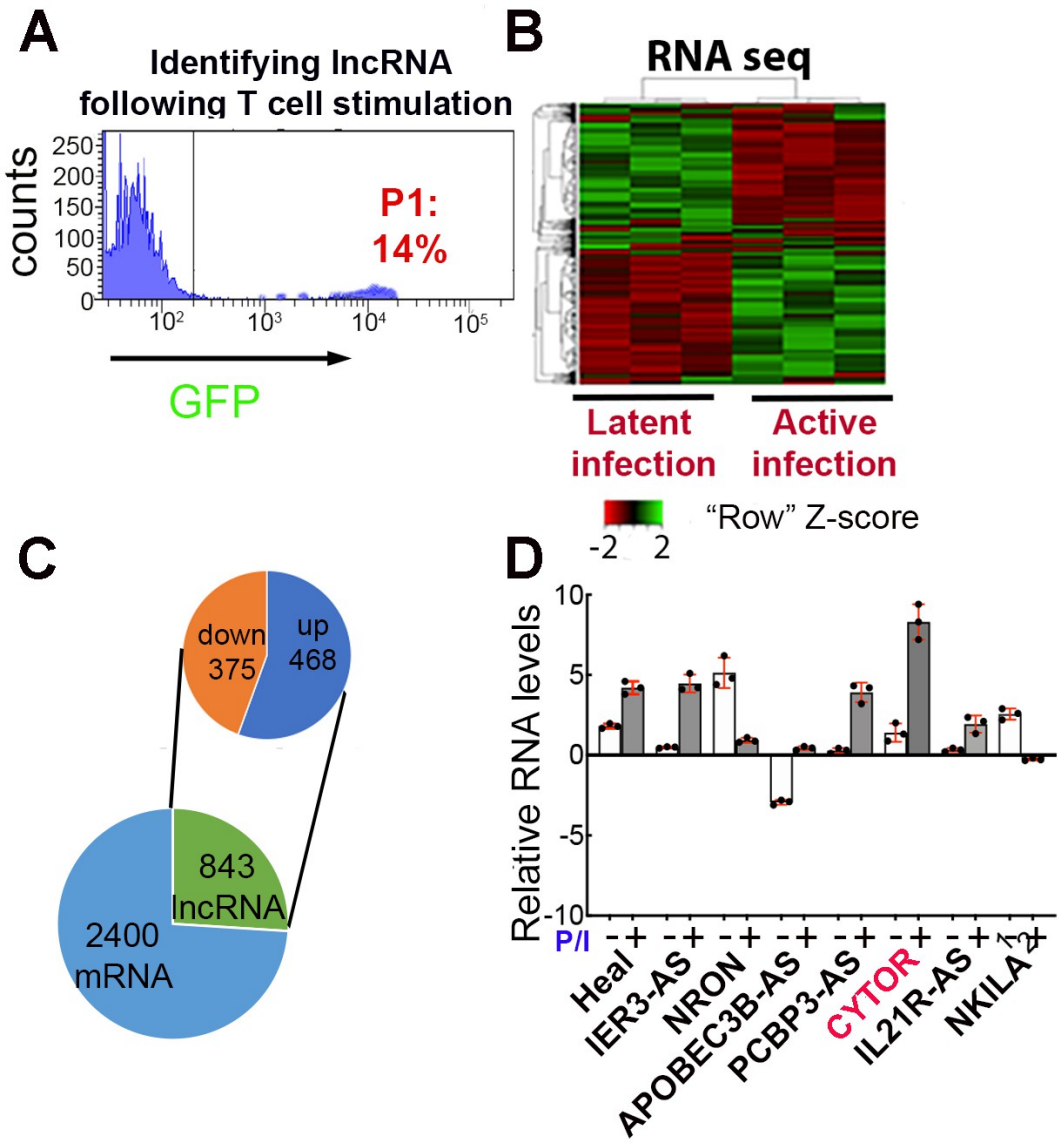
Identifying host ncRNAs that are differentially expressed in Jurkat T cells that carry active or latent HIV following T-cell activation. **(A)** FACS histogram analysis of PMA/Ionomycin (P/I)-stimulated J-Lat 6.3 cells. GFP(+) cells carrying active HIV (P1 region) were sorted from GFP (-) cells carrying latent HIV (GFP-). Cells were sorted and sent to RNA-Seq (n = 4). **(B)** Heatmap of differential transcript expression pattern (FC≥ a 2-fold change and above between cells carrying active *versus* latent HIV with an adjusted p value of ≤0.05. **(C)** Pie chart corresponding to the numbers of differentially expressed mRNAs and lncRNAs, up and downregulated in cells where HIV was reactivated. **(D) RNA levels of selected ncRNA in primary CD4+ T cells**. Analysis of expression levels of selected ncRNA based on the RNA-Seq analysis in primary CD4+ T cells that were either under resting conditions (-) or stimulated with P/I (+). RNA levels were analyzed by RT-qPCR. Data were normalized to *gapdh* levels. Data are from 2 healthy donors.

In search for novel host regulators of HIV gene expression and viral latency, we employed RNA-Sequencing analysis to monitor changes in the transcriptome of Jurkat-derived J-Lat 6.3. These cells carry a transcriptionally repressed intact copy of HIV-1 proviral DNA with a GFP reporter under the control of the HIV promoter that is inserted in the *nef* gene. As expected, in response to T-cell stimulation, HIV gene expression in J-Lat 6.3 cells was enhanced, as exhibited by elevated expression levels of GFP [41]. J-Lat 6.3 cells were stimulated with the PKC activator PMA and Ionomycin (P/I), which potently activate CD4+ T lymphocytes. Stimulated J-Lat 6.3 cells were then sorted by FACS based on their HIV-GFP expression and divided into two distinct populations: Stimulated cells that expressed HIV genes (GFP+; P1) and stimulated cells that carried latent provirus (GFP-) (Fig 1A). RNA from both cell groups was isolated, and libraries were generated for transcriptome analysis by next generation sequencing (RNA-Seq). As expected, a pronounced change in cellular gene expression, including mRNAs, miRNAs, snoRNAs, snRNAs and lncRNAs was observed in stimulated cells that expressed active HIV or latent HIV (Fig 1B). Subsequent RNA-Seq from HIV expressing cells that carry active (GFP+) or latent (GFP-), indicated different transcriptional profiles in cells where HIV is activated *versus* cells where the virus remained latent. A total of 3490 annotated transcripts were identified whose expression was changed in cells that carried transcriptionally active HIV relative to latent HIV. Of these, 2400 transcripts corresponded to protein-coding genes, while 843 were lncRNAs. Upon T-cell stimulation, 468 lncRNA transcripts were upregulated (enriched in cells expressing active HIV; GFP+), and 375 were downregulated (enriched in cells carrying latent HIV provirus; GFP-) (Fig 1C). We further assessed the relative expression levels of the highly ranked lncRNAs in CD4+ T primary T cells by RT-qPCR. Analysis was performed under resting or stimulating conditions of primary CD4+ T cells. For most tested lncRNAs, a shift in expression levels was confirmed when comparing primary CD4+ T cells under resting or stimulated conditions, where HIV was latent or active, respectively (Fig 1D). Notably, ncRNAs with reported effects on HIV replication and latency, including HEAL [38] and NRON [37] were identified via our RNA-Seq analysis, demonstrating the potential of this screening approach (Fig 1D). Also indicated are ncRNAs that are currently under investigation like IL21R-AS; PCBP3-AS; APOBEC3B-AS; IER3-AS (Fig 1D). Similarly, mRNAs genes, including HSP90 [42], ESR-1 [43], and IFI16 [44], that were previously reported to control HIV replication were also identified by our screening approach (S1 Table; GSE254771). Finally, we confirmed surface expression of CD25 and CD69 activation markers in stimulated primary CD4+ T cells that were infected with HIV_GKO_ and carry active or latent HIV (S1 Fig).

Among the lncRNAs that were strongly induced upon T cell stimulation, we focused our work on Cytoskeleton Regulator RNA (CYTOR)—also known as lincRNA00152. Elevated RNA levels of CYTOR upon T cell stimulation were confirmed in J-Lat 6.3 and in primary CD4+ T cells (Fig 1D). To monitor the effects of HIV infection on CYTOR RNA levels, Jurkat T cells were infected with HIV and levels of CYTOR RNA were determined by RT qPCR relative to non-infected cells. Our analysis confirmed that CYTOR RNA levels were not affected by HIV infection (S2 Fig). CYTOR is an intergenic 828 nucleotide lncRNA located on chromosome 2p11.2. It is highly conserved in primates and rodents but less so in lower organisms. CYTOR is mainly present in the cytoplasm. However, previous reports show that it is also localized to the nucleus. Within the nucleus, CYTOR functions as an oncogene and is upregulated in multiple human malignancies [45]. CYTOR also acts as an “endogenous sponge” for several micro-RNAs by binding to them, inhibiting their activity, and promoting malignancy. Interestingly, CYTOR reportedly regulates cellular actin dynamics and cytoskeletal reorganization in fibroblasts by controlling the expression of genes of the actin polymerization machinery [46]. However, the functional importance of CYTOR in CD4 T cells and in the context of HIV infection has not been studied.

### CYTOR activates HIV gene expression and affects latency reversal

**Fig 2:**
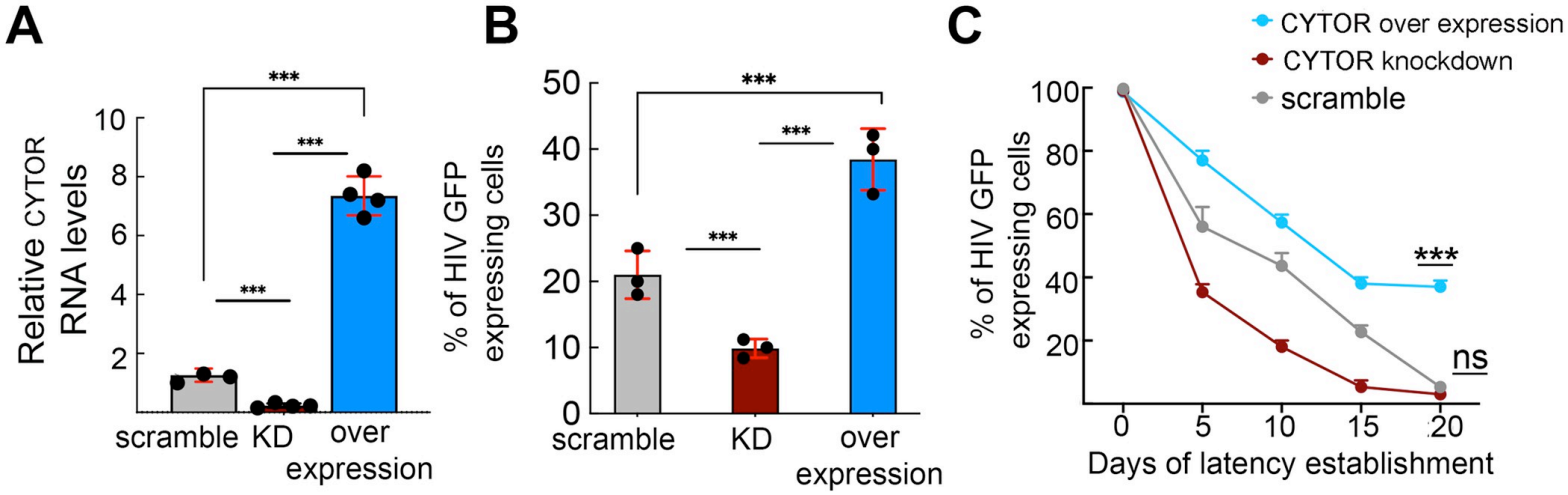
Effects of CYTOR expression on HIV gene expression and latency establishment. (**A**). **Modulation of CYTOR RNA levels in J-Lat 6.3 cells**. RT-qPCR analysis measuring CYTOR RNA levels in J-Lat 6.3 T cells, where CYTOR expression is knockdown (KD; red bar) or overexpressed (light blue bar). RNA levels were normalized to *gapdh* and presented relative to control cells expressing scrambled shRNA (grey bar). Statistical significance is based on calculating ±SD of data points from four independent experiments using two-way ANOVA. ***p≤0.05. (**B**) **Effects of CYTOR on HIV gene expression**. FACS quantification analysis of the percentage of cells that express HIV-GFP in P/I stimulated J-Lat 6.3 cells expressing control scramble shRNA (grey bar), or in which CYTOR was overexpressed (blue bar) or knockdown (KD; red bar). Statistical significance is based on calculating mean ± SD from three independent experiments using two-way ANOVA. ***p≤0.05. (**C**) **Kinetics of latency establishment in the context of CYTOR expression**. 2D10 latency model Jurkat T cells that carry a mini-*Tat-Rev* GFP under the regulation of the HIV LTR promoter and express either scrambled shRNA (grey), CYTOR KD (red), or cells over-expressing CYTOR (blue) were reactivated and sorted to obtain a pure cell population that expresses GFP. GFP expression was then followed over time as a measurement of entry into latency. Statistical significance is based on calculating mean ± SD from three independent experiments using two-way ANOVA. ***p≤0.05 and ns: not significant.

We next conducted gain and loss-of-function studies in J-Lat 6.3 T cells to determine the role of CYTOR in regulating HIV gene expression. To achieve CYTOR over-expression, cells were transduced with a lentivirus that drives the expression of CYTOR—exons 1, 4, and 5, the most abundantly expressed form in humans [47]. Following antibiotic selection, resistant J-Lat 6.3 T stable cells were subjected to RT-qPCR and exhibited a significant increase in CYTOR RNA levels relative to control cells (Fig 2A; blue bar versus grey bar). Reducing CYTOR expression (knockdown; KD) was also achieved by transducing J-Lat 6.3 T cells with a lentivirus encoding a CYTOR-targeting small-hairpin RNA (shRNA), resulting in a significant decrease of CYTOR RNA levels relative to control cells, expressing a scrambled shRNA as measured by RT-qPCR (Fig 2A; red bar versus grey bar). Parallel FACS-based analysis of GFP expression in HIV-infected J-Lat 6.3 cells, as a measure of viral gene transcription, revealed that in the absence of T-cell stimulation, no effects on HIV gene expression were observed upon modulation of CYTOR expression. However, following T cell stimulation with P/I, CYTOR over-expression led to a relatively small 2-3-fold increase of HIV GFP expression over control cells (Fig 2B; compare blue to grey bars). In contrast, depletion of CYTOR led to a 5-fold decrease in HIV gene expression over control cells (Fig 2B; compare red to grey bars). HIV GFP expression in control cells expressing scrambled shRNA was unaffected (Fig 2B; grey bar).

We also followed the establishment of HIV latency post-T cell activation, documenting HIV-GFP expression in control 2D10 cells that expressed scramble shRNA or in cells where CYTOR expression levels were modulated. Like J-Lat 6.3 cells, 2D10 cells serve as a Jurkat-based latency cell model that carries a minimal *Tat-Rev* cassette in the context of a GFP reporter under the regulation of the HIV promoter. Upon T-cell stimulation of control 2D10 Jurkat cells, we confirmed that the expression of HIV-GFP was significantly induced. Control and CYTOR-modulated stimulated 2D10 Jurkat cells were sorted based on their HIV-GFP expression, obtaining a relatively pure cell population with 100% HIV-GFP expression levels. We then monitored latency establishment by following GFP expression in the context of control or CYTOR-modulated cells (Fig 2C). Our FACS analysis revealed that lower CYTOR levels were associated with a rapid establishment of latency relative to control cells (Fig 2C; grey versus red lines). Conversely, CYTOR-over-expression enhanced latency reversal, as determined by the elevated levels of HIV-GFP expression that remained relatively high for an extended period following T cell stimulation (Fig 2C; blue line). These results suggest that CYTOR expression activates HIV gene expression, significantly reversing latency in 2D10 cells.

### CYTOR activates HIV gene expression in stimulated primary CD4+ T cells

**Fig 3:**
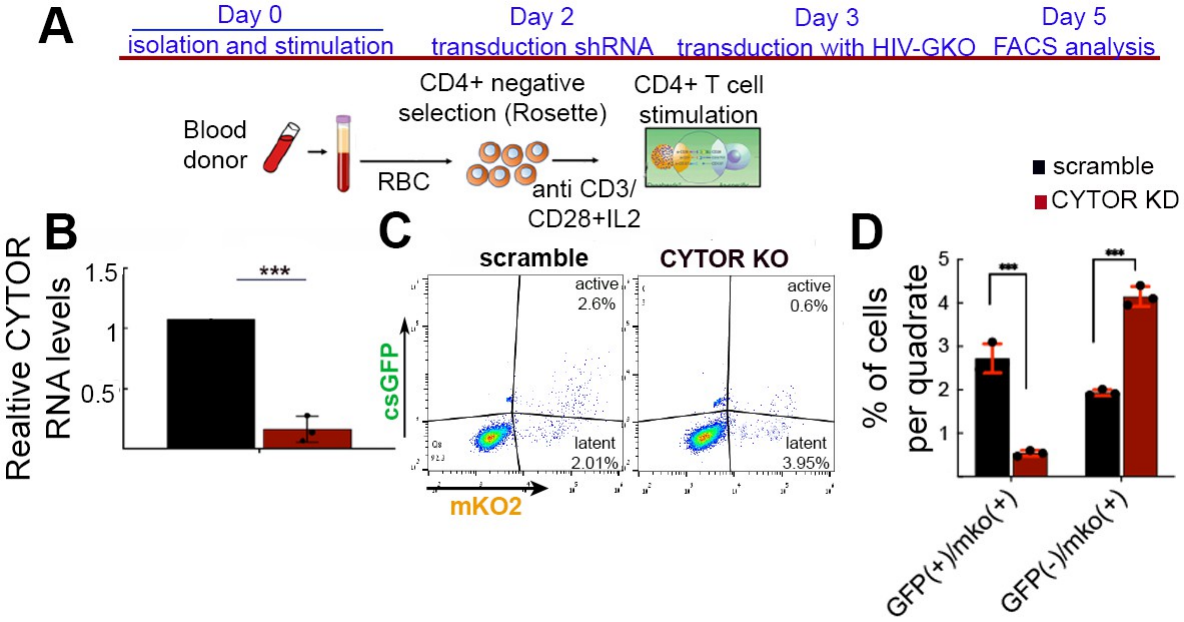
CYTOR depletion in primary CD4+ T cells suppresses HIV infection and promotes latency establishment. (A). Experimental workflow overview for isolating primary CD4+ T cells. See methods for a detailed description. The figure was generated by Biorender. (B). Depletion of CYTOR in stimulated primary CD4+ T cells using lentivirus encoding CYTOR shRNA. Data were measured by RT-qPCR, normalized to GAPDH, and presented relative to cells expressing scrambled shRNA—set to 1. Statistical significance is based on calculating mean ± SD from three independent experiments using two-way ANOVA. ***p≤0.05; n = 3. (C). FACS analysis presenting effects of CYTOR knockdown (KD) on HIV_GKO_ infection in primary CD4+ T cells. Cells were stimulated and then transduced with HIV_GKO_ before being analyzed by FACS for mKO2 and GFP expression. (D). Quantification of quadrate percentage from three independent experiments of FACS analysis for HIV_GKO_ transduction in CD4+ primary T cells, where CYTOR is KD. Statistical significance is based on the calculation of mean ±SD from three independent experiments (n = 3) using Two-way ANOVA. ***p≤0.05.

Next, we shifted our analysis to CD4+ primary T cells isolated from healthy donors and the natural target cells for HIV infection. Depletion of CYTOR in primary human CD4+ T cells was achieved by first stimulating purified cells with anti CD3/CD28 beads and IL2 and then transducing them with a lentivirus encoding a CYTOR-specific shRNA. Lentivirus driving the expression of scrambled shRNA was used as a control (Fig 3A; n = 3). RT qPCR confirmed a significant decrease in CYTOR expression RNA levels relative to control cells that expressed the scramble shRNA (Fig 3B). The next day, CYTOR-depleted CD4+ primary stimulated cells (KD) or control cells were transduced with HIV_GKO_, which codes for a codon-optimized GFP reporter under the control of the HIV-1 promoter and in the context of expression of all viral proteins, and a mKO2 reporter under the control of the constitutive promoter EF10 α [48]. HIV_GKO_ transduction can be analyzed by FACS two days later, monitoring parallel transduction efficiency (mKO2+), as well as HIV gene expression (GFP+). Upon transduction of stimulated primary CD4+ T cells, KD of CYTOR led to decreased HIV gene expression, as monitored by reduced levels of HIV-GFP-expressing cells. Conversely, the proportion of cells that expressed EF10 α -mKO2 was slightly elevated in control or CYTOR KD-expressing cells, implying that transduction efficiencies were not affected due to CYTOR depletion but rather specifically drove HIV into a latency state (Fig 3C and 3D).

### CYTOR is localized to the nucleus, binds to the HIV promoter, and modifies RNA polymerase II phosphorylation state and histone landscape

**Fig 4:**
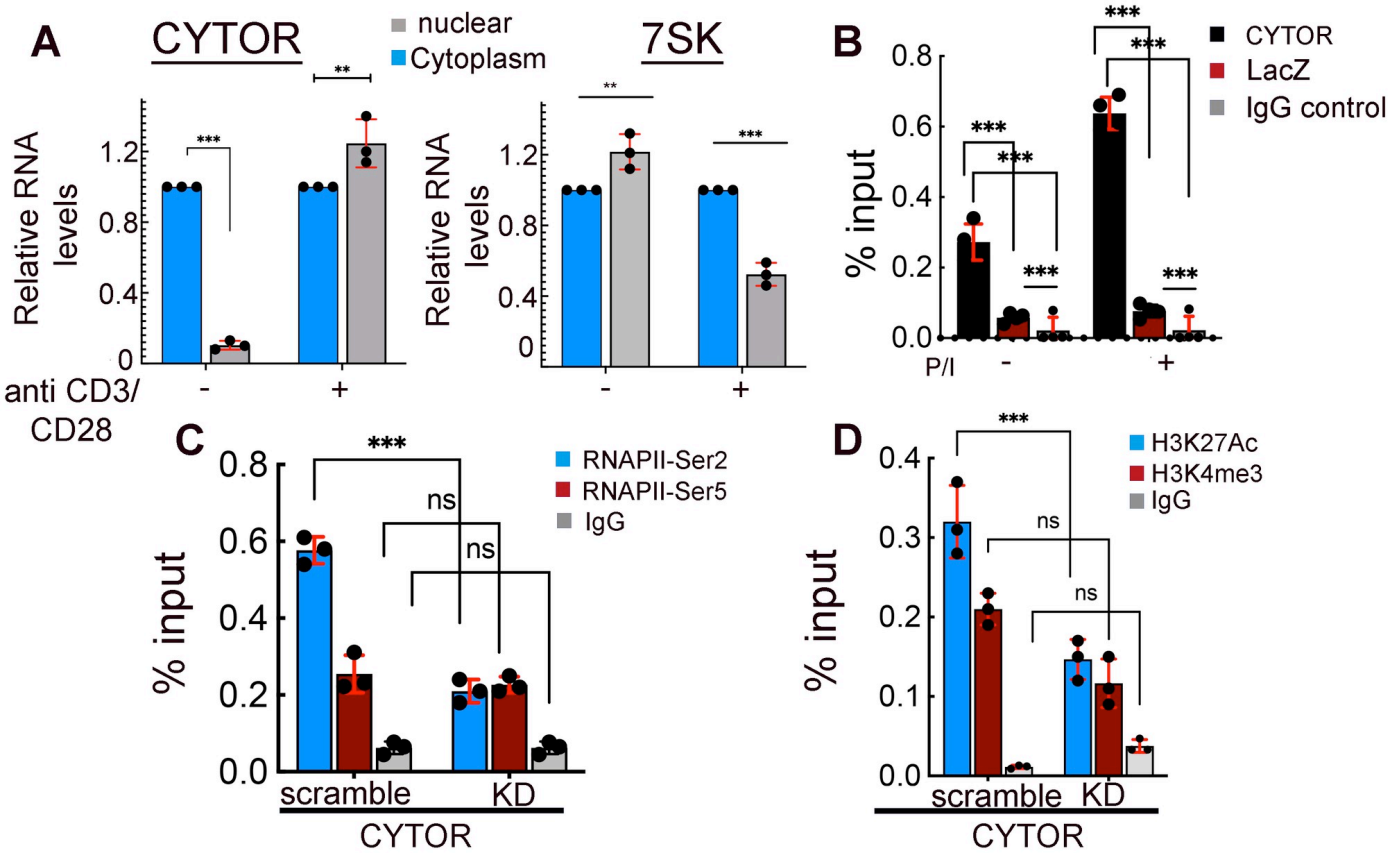
CYTOR is localized in the nucleus, binds to the HIV promoter, and modifies RNAPII CTD phosphorylation state and histone landscape. (**A**). **CYTOR is localized to the nucleus and its levels are elevated upon T-cell stimulation**. Resting or stimulated primary CD4+ T cells were subjected to cell fractionation, separating the samples into a nuclear fraction (grey bar) or cytoplasmic fraction (light blue bar). Samples were then subjected to RT-qPCR and monitored for CYTOR or 7SK ncRNA levels. Data were normalized to 7SL RNA in each of the cellular fractions and conditions. Data are presented relative to cytoplasmic fraction in each condition—set to 1. **(B). ChIRP-qPCR analysis for CYTOR binding to the HIV promoter**. CYTOR-specific (black bar) or control lacZ (red bar) antisense biotinylated probes were incubated with lysates isolated from unstimulated or P/I stimulated J-Lat 6.3 cells. Biotinylated RNA was pulled down with streptavidin beads, and following washing, associated DNA was eluted and analyzed by qPCR with primers for the HIV promoter. Statistical significance was calculated between the two probes and between unstimulated and stimulated states. IgG served as a non-specific antibody for IP control (grey bar). The analysis is based on calculating mean ± SD from three independent experiments using two-way ANOVA. ***p≤0.05. **0.05≤p≤ 0.1; n.s—not significant. (**C, D**) **CYTOR affects the phosphorylation state of RNAPII CTD and histone landscape**. ChIP qPCR analysis in control or CYTOR KD J-Lat 6.3 cells. ChIP material from cells was immune-precipitated (IP) with antibodies targeting RNAPII-Ser2P or RNAPII Ser5P (**C**); or for H3K4me3 and H3K27Ac histone activation marks (**D**). IP fraction was analyzed for enrichment of the indicated modifications on the HIV promoter by qPCR with specific primers. Non-specific IgG served as a control (grey bar). Percentage of input are means ±SD; n = 3; *** p≤0.05 calculated between scrambled and KD cells for each antibody. n.s—not significant.

Towards direct effects of CYTOR on HIV gene transcription, this would require its localization within the cell nucleus. We therefore monitored the subcellular distribution of CYTOR lncRNA between the nucleus and cytoplasm in resting or activated conditions of primary CD4+ T cells by cell fractionation and subsequent RT-qPCR analysis (Fig 4A). Levels of CYTOR RNA were compared to those of the abundant 7SK lncRNA, which is known to interact with inactive P-TEFb. RNA levels were normalized to the 7SL RNA, which does not bind to the HIV promotor and is commonly used as a specificity control for these experiments [33]. Our analysis showed that CYTOR is localized to the cytoplasm and the nucleus. Notably, upon T cell stimulation, levels of nuclear CYTOR were elevated relative to the levels of nuclear 7SK, which were decreased (Fig 4A). To further extend our understanding of the mechanism by which CYTOR activates HIV gene expression, we tested whether CYTOR binds to the HIV promoter, thereby regulating HIV gene transcription. We monitored CYTOR occupancy on the HIV promoter by employing Chromatin Isolation by RNA Purification (ChIRP) analysis in J-Lat 6.3 cells. In vitro-transcribed biotinylated CYTOR RNA was synthesized, purified, and then incubated with ChIP material isolated from unstimulated or P/I stimulated HIV J-Lat 6.3 cells. RNA-protein complexes were then specifically pulled down with streptavidin beads, and pulled down levels of CYTOR on the HIV promoter were monitored by RT-qPCR with specific primers that target the viral promoter [49] (Fig 4B). Our analysis showed that CYTOR binds to the HIV promoter even in unstimulated conditions. Significantly, CYTOR occupancy on the viral promoter was further elevated following T-cell stimulation (Fig 4B). CYTOR occupancy on gene promoters was also demonstrated for cellular genes that are known to be regulated by P-TEFb, such as *NF-κB*, *IL21Ra*, *myc*. Of note, the binding of CYTOR to HIV downstream reverse transcriptase sequences was not observed, suggesting that the specificity of CYTOR within the HIV genome lies within the HIV promoter (S3 Fig).

To further obtain insights into the mechanisms of action of CYTOR, we performed Chromatin immunoprecipitation (ChIP) qPCR from J-Lat 6.3 cells, where CYTOR expression was manipulated. We monitored the levels of phosphorylated C-terminal domain (CTD) of RNA Polymerase II (RNAPII) at Ser2 (Ser2P) or Ser5 (Ser5P) residues on control or CYTOR KD expressing cells, using specific antibodies that target the CTD phosphorylation states of RNAPII (Fig 4C). CDK9/P-TEFb phosphorylates Ser2 and marks RNAPII pause-release and elongation of transcription [50–52]. CDK7/TFIIH phosphorylates Ser5P on the CTD and catalyzes transcription initiation and promoter clearance. Our analysis demonstrated that in the context of CYTOR depletion, levels of Ser2P on the HIV promoter were decreased without affecting those of Ser5P, implying the involvement of P-TEFb in CYTOR-mediated HIV gene activation (Fig 4C). In addition, ChIP-qPCR was employed using antibodies that target the histone activation markers, H3K27Ac or H3K4me3. Our analysis confirmed that CYTOR mediates its activation properties by modifying the histone landscape around the HIV. Upon CYTOR depletion, levels of these histone activation markers were reduced (Fig 4D). These results further imply that CYTOR activates HIV gene expression via P-TEFb, affecting transcription elongation.

### CYTOR associates with P-TEFb

**Fig 5:**
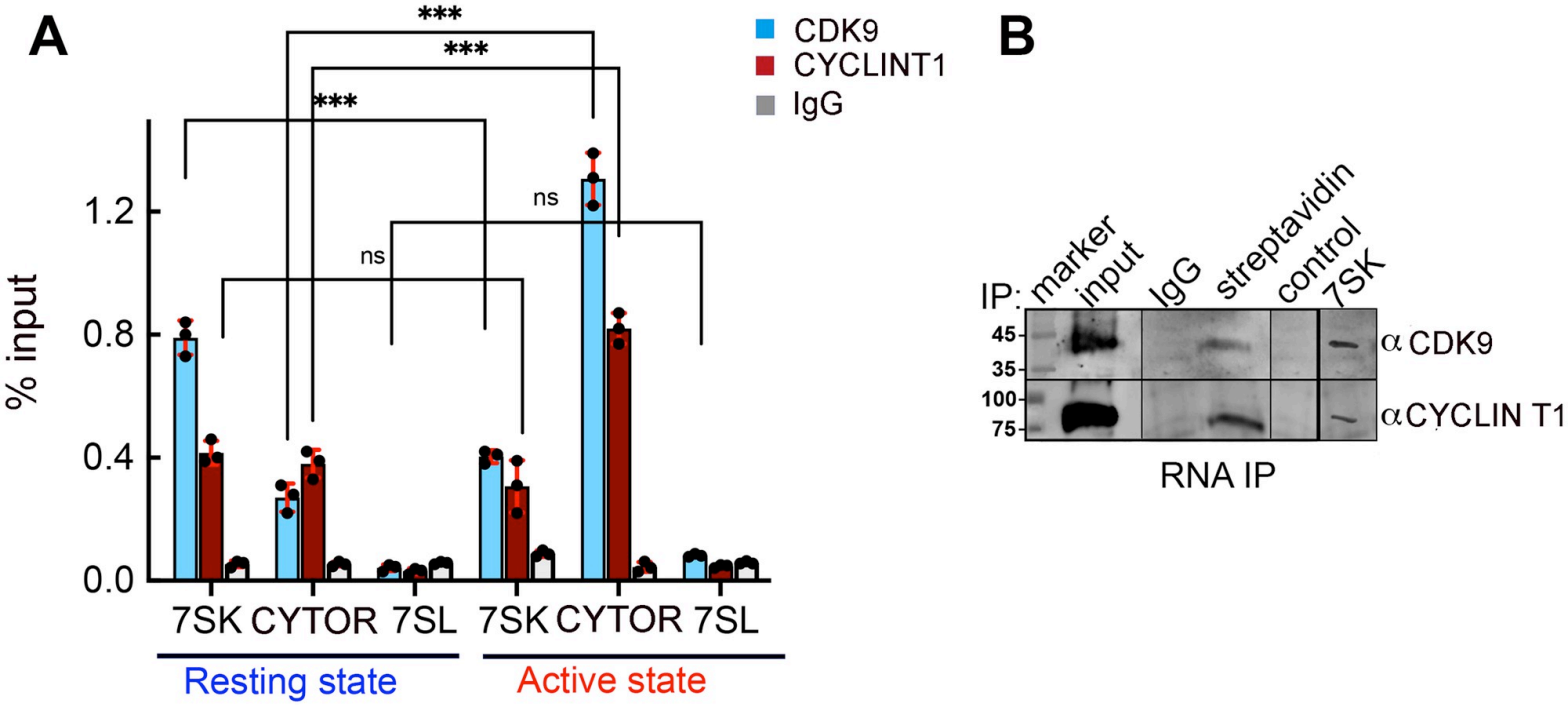
CYTOR associates with P-TEFb in cells. (**A**) RIP analysis demonstrates **the association of CYTOR with P-TEFb**. Isolated ChIP material from resting or stimulated J-Lat 6.3 CD4+ T cells was subjected to immune precipitation with antibodies targeting CDK9 or CYCLIN T1 of P-TEFb, followed by RT-qPCR with primers for the relevant lncRNA (7SK or CYTOR). Non-specific IgG served as a control for the IP step. 7SL ncRNA served as a control for an RNA that does not associate with P-TEFb and, therefore, not precipitated with CDK9 or CYCLIN T1 antibodies. Statistical significance is based on the calculation of mean ±SD from three independent experiments using two-way ANOVA. ***p≤0.05. ** 0.05≤p≤0.1; ns: not significant. (**B**) **CYTOR associates with P-TEFb in cells**. RNA pull-down followed by western blotting where lysates from J-Lat 6.3 cells were incubated with an in-vitro transcribed biotinylated CYTOR anti-sense probe and reactions were pulled down with streptavidin beads. Eluted RNP complexes were subjected to western blotting with indicated antibodies. Non-specific IgG served as a control for non-relevant IgG. Scramble RNA served as RNA that does not associate with P-TEFb. 7SK probe confirmed association with P-TEFb. Input is 5% of the total cell lysate [63].

To expand the above results on the mechanism by which CYTOR enhances HIV gene expression, we employed RNA-precipitation (RNA-IP; RIP) followed by RT-qPCR in J-Lat 6.3 cells under resting or stimulated conditions (Fig 5). As our above results indicate that CYTOR promotes the Ser2 phosphorylation on the CTD, which is mediated by CDK9, we monitored CYTOR association with P-TEFb. Lysates isolated from nuclei from resting or stimulated J-Lat 6.3 cells were incubated with antibodies that target CDK9 or CYCLIN T1, and samples were IP followed by RT-qPCR to detect CYTOR RNA levels by using specific primers. We show that upon T cell stimulation, the levels of CYCLIN T1 and CDK9 that were associated with CYTOR RNA increased. As expected, levels of 7SK that are associated with P-TEFb were reduced upon T cell stimulation. We also followed the association of P-TEFb with 7SL, which served as control. As expected, P-TEFb was not associated with 7SL in each of the tested conditions. These results indicate that CYTOR associates with P-TEFb in cells (Fig 5A).

Next, we performed RNA pull-down experiments combined with western blotting to detect P-TEFb subunits (CYCLIN T1/CDK9) that are associated with CYTOR. Lysates from J-Lat 6.3 cells were incubated with an *in-vitro* transcribed biotinylated anti-sense CYTOR probe, and reactions were pulled down with streptavidin beads. Eluted RNP complexes were then subjected to western blotting with antibodies that target CYCLIN T1 or CDK9, demonstrating the association of CYTOR lncRNA with P-TEFb within cells. Non-specific IgG was used as a specificity control for the IP step, while a non-specific scramble RNA probe served as a control for RNA-protein association. In addition, a 7SK RNA probe confirmed the association with P-TEFb (Fig 5B). These results establish that CYTOR binds to the HIV promoter and suggest that its activation effects are mediated by association with P-TEFb.

### CYTOR regulates actin dynamics in response to T cell activation

**Fig 6:**
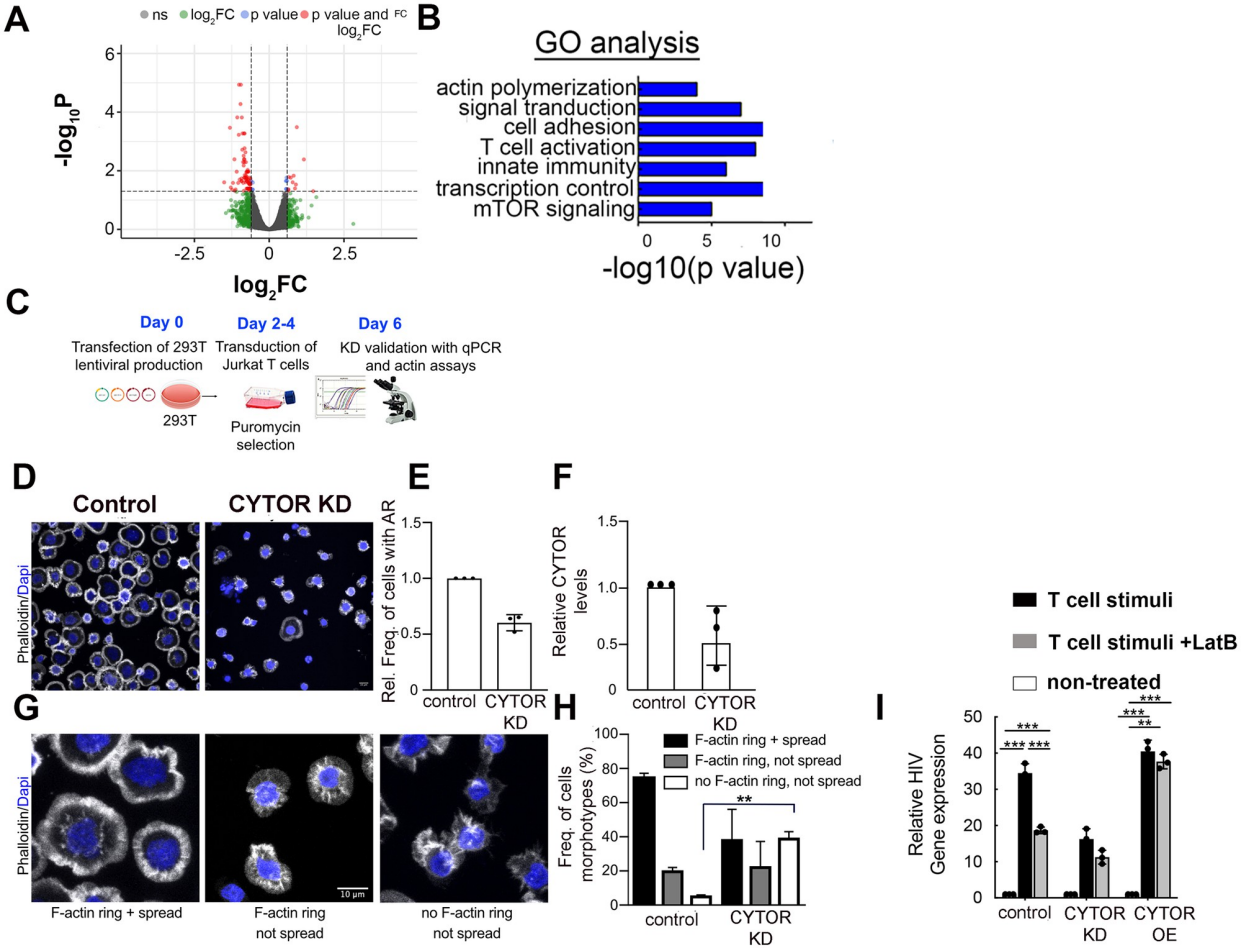
CYTOR is required for TCR-induced cytoplasmic actin remodeling. (**A)**. Volcano plot of the expression pattern of genes from an RNA-Seq analysis upon CYTOR depletion following T cell stimulation. -Log_10_ P is shown on the y-axis, and Log_2_FC is on the x-axis. RNA was isolated from 3 biological replicates (n = 3). The fold of change cutoff is defined as FC ≥2. FDR of p≤0.05 was used as a cutoff for significance. (**B**). Gene Ontology analysis for enriched CYTOR gene targets. For enrichment analysis, the DAVID program was employed to identify enriched pathways and terms associated with the selected genes. **(C)**. Experimental flow for microscopy-base analysis of cell morphology and formation of F-actin rich structures. The figure was generated by Biorender. **(D)**. Representative confocal images of F-actin organization for control and CYTOR KD Jurkat cells after contact with anti-CD3/28 coated surfaces. Cells were stained with fluorescent phalloidin and DAPI to visualize F-actin and cell nuclei. Shown are merged images of both channels, scale bar = 10 μM). **(E)**. Relative frequency of cells with circumferential F actin ring (AR) in control or CYTOR KD cells with proper cell spreading and circumferential F-actin relative to control cells (mean± SD, 100 cells per experiment/condition, n = 3). **(F)**. Relative CYTOR RNA levels in CYTOR KD Jurkat cells relative to control cells of the cells analyzed in (E). **(G)**. Representative images of the different morphotypes observed for Jurkat cells after anti-CD3/28 surface stimulation (analyzed as in D), **(H)**. Quantifying the frequency of the morphotypes defined in (G) for control and CYTOR KD Jurkat cells (mean± SD, 100 cells per experiment/condition, n = 3). ** 0.05≤p≤0.1. **(I). Inhibition of actin remodeling disrupts HIV gene expression upon T cell activation**. 2D10 cells carrying an integrated HIV-GFP provirus where CYTOR expression was either depleted or over-expressed were treated with an actin polymerization inhibitor, Latrunculin B (LanB) for 1 hour, followed by T cell stimulation with anti-CD3/CD28 for an additional 3 hours. Cells were harvested 24 hours later, and the percentage of cells expressing HIV GFP was monitored by FACS. Data are presented as fold of activation relative to untreated cells and activated with the indicated T cell activator. Statistical significance is based on calculating mean ± SD from three independent experiments using two-way ANOVA. ***p≤0.05. ** 0.05≤p≤0.1.

Since CYTOR has been previously recognized as a regulator of cytoskeleton-regulating genes in fibroblasts [46], we assessed if it could also affect HIV gene expression by indirect mechanisms through regulation of its downstream targets. For this, we performed RNA-Seq analysis in stimulated primary CD4+ T cells, where CYTOR expression was depleted or over-expressed (n = 3). CYTOR modulation of expression did not affect the activation state of cells as monitored by staining with T cell activation markers (S4 Fig). Analyzing changes in the cellular transcriptome of stimulated CD4+ T cells upon depletion of CYTOR revealed a modest change in the cell gene expression program (S2 Table). Additional gene GO analysis identified significant enrichment scores in various cellular pathways, including those of gene expression, signal transduction as well as actin dynamics and T-cell activation (Fig 6A and 6B).

T cell activation elicits complex and highly dynamic signaling cascades that ultimately lead to the activation of transcription factors, including NF-κB and NF-AT, to increase the expression of T cell receptor target genes [53,54]. The involvement of these transcription factors in HIV gene expression, at least in part, explains the beneficial effects of T cell activation on HIV gene expression [5]. Since many of the downstream signaling events elicited by TCR engagement depend on the immediate polymerization of cortical actin, we tested if CYTOR affects the actin polymerization response to TCR engagement of Jurkat T cells. Scramble control or CYTOR KD Jurkat cells were placed on a cell stimulatory surface coated with anti-CD3/CD28 antibodies, fixed, and stained for F-actin. Control cells displayed the typical cell spreading and formation of circumferential F-actin-rich rings (actin ring; AR) (Fig 6C, 6D, 6E and 6F). Although CYTOR expression was only moderately reduced in KD cells (Fig 6F), fewer cells responded to TCR stimulation (approx. 40% less cells with AR in CYTOR KD than in control cells; Fig 6E). Detailed analysis of the different cell morphologies revealed that the CYTOR KD particularly resulted in a significant increase in the fraction of cells that were unable to both spread as well polymerize actin into an F-actin ring in response to T cell activation. In contrast, the frequency of cells that failed to spread despite efficient actin polymerization was unaffected (Fig 6H). Increasing CYTOR levels by overexpression did not further increase the frequency of cells that formed ARs, did not alter the morphology of F-actin structures formed in response to TCR activation, and did not result in the formation of ARs in the absence of TCR stimulation (S5 Fig). We conclude that CYTOR is an important regulator of TCR-induced actin polymerization in CD4+ T cells, but its normal endogenous expression levels are not limiting for this response.

### TCR-mediated latency reversal requires actin polymerization

To assess whether TCR-induced actin remodeling affects HIV gene expression in our experimental system, we measured the induction of HIV gene expression by TCR engagement in 2D10 cells, a CD4+ T cell line that carries a latent GFP cassette under the control of the LTR promoter. Experiments were performed in the absence or presence of the actin polymerization inhibitor, Latrunculin B—an inhibitor that interferes with actin polymerization and is reversible upon washout [55] (Fig 6I). Stimulation with anti-CD3/anti-CD28 resulted in a marked induction of GFP expression and, as observed before, silencing CYTOR expression reduced this induction. Notably, interfering with actin polymerization during the first 3 hours of TCR stimulation in control cells limited the induction of HIV gene expression to the levels observed upon CYTOR KD, and interference with actin dynamics in CYTOR KD did not result in an additional reduction of GFP expression. Finally, overexpressing CYTOR rendered the TCR-mediated induction of GFP expression insensitive to Latrunculin B (Fig 6I) [55]. Together, these results reveal that the regulation of host cell actin dynamics is necessary but not sufficient for the regulation of gene expression of latent HIV.

## Discussion

In search of regulators of HIV latency, we profiled changes in the expression of ncRNAs by employing RNA-Seq analysis in resting and stimulated HIV-infected J-Lat 6.3 T cells, comparing RNA expression levels in cells that carry active HIV (GFP+) or latent HIV (GFP-). Our analysis show that different transcriptional profiles exist in cells where HIV is activated versus cells where it remains latent. CYTOR lncRNA was identified as one of these RNAs, and its expression is elevated upon T cell stimulation, where HIV is active. These observations were further confirmed in primary CD4+ T cells (Fig 1). Functional analyses show that following T cell stimulation, over-expression of CYTOR activates HIV gene expression, while its depletion inhibits viral gene expression. Significantly, upon T cell stimulation, depletion of CYTOR promoted entry of HIV into a latent state, while its over-expression delayed entry into latency and enhanced latency reversal (Fig 2). Effects of CYTOR on HIV infection and latency establishment were also confirmed in stimulated primary CD4+ T cells (Fig 3). We are aware that the model of stimulated CD4+ primary cells does not recapitulate the actual state of the reservoir, which is mainly comprise of resting CD4+ T cells that do not support HIV infection. As this is a limitation of the current study, we are trying to adopt a recently developed gene editing approach to lncRNAs to deplete CYTOR in this unique cell population and monitor the effects of latency kinetics without altering its activation [56]. Mechanistically, our observations show that CYTOR directly binds to the HIV promoter and enhances the phosphorylation of the Ser2 CTD of RNAPII through association with P-TEFb to activate viral gene expression (Figs 4 and 5). Changes in histone activation marks around the viral promoter in CYTOR-depleted cells also imply that CYTOR activates the proviral gene expression (Fig 4).

**Fig 7:**
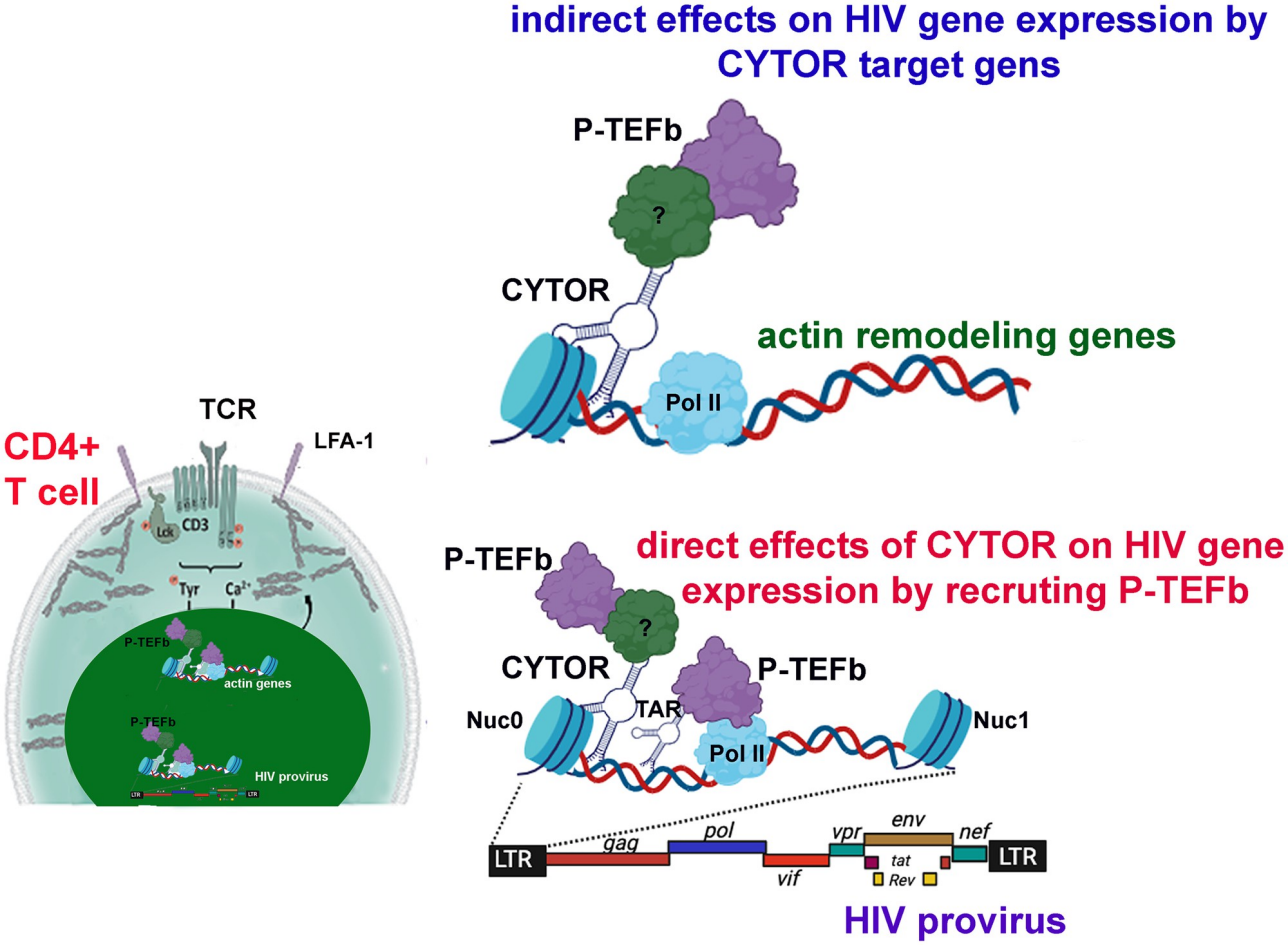
A working model for CYTOR functions. Following T cell activation, levels of CYTOR are elevated in the nucleus. CYTOR is recruited to the HIV promoter and binds to P-TEFb, leading to the activation of viral gene expression. Cellular genes regulated by CYTOR include actin remodeling genes that promote actin polymerization and the indirect activation of HIV gene expression.

In addition to the direct effects of CYTOR on HIV gene expression, we also demonstrate that CYTOR controls global gene expression. CYTOR is recruited to other gene promoters that are regulated by P-TEFb, like *myc*, NF-κB, and *IL2Ra* (S3 Fig). Among the identified enriched pathways that potentially are regulated by CYTOR are those that are involved in actin dynamics. Consistently, reduced levels of CYTOR expression are associated with reduced polymerization of cortical actin in response to TCR engagement (Fig 6). In turn, elevated levels of CYTOR do not further increase actin polymerization in response to T cell stimulation and cannot induce morphological responses of T cells in the absence of stimulation (S5 Fig). Thus, CYTOR is an important regulator of TCR-induced actin polymerization in T cells. However, its normal endogenous expression levels are sufficient for a proper response. To test a mechanistic link between actin remodeling, CYTOR levels, and HIV gene expression, we inhibited actin dynamics with specific inhibitors (Fig 6I). Effects of inhibition of actin polymerization phenocopied the effect of CYTOR depletion on HIV gene expression, suggesting that CYTOR may affect HIV gene expression by the regulation of genes that control cellular actin dynamics (Fig 6I). Accordingly, we propose a model where CYTOR exerts its effects on global gene expression and promotes HIV gene expression by both direct and indirect effects (Fig 7). CYTOR directly binds the HIV promoter and recruits the elongation transcription machinery to enhance RNAPII CTD phosphorylation and deposition of active histone markers around the HIV promoter, ultimately activating HIV gene expression. Indirectly, CYTOR controls gene targets that regulate actin dynamics in the nucleus and at the plasma membrane to optimize the response to T cell activation, presumably via the regulation of cellular gene expression.

Like CYTOR, other lncRNAs have been reported to occupy the HIV promoter and modulate its activity at either transcriptional or posttranscriptional levels [57]. Most act as scaffolds that associate with other transcriptional activators or repressors to control HIV gene expression [35–39,58–60]. In the case of CYTOR, its effects on gene expression occur by recruiting the transcription elongation machinery to activate gene expression, either from the viral promoter or other cellular promoters. It will be essential to identify other partners that are associated with CYTOR lncRNA and control HIV promoter activity. As we also aim to dissect the role of CYTOR in gene expression control, specifically for HIV gene regulation, it will be essential to define how events within the nucleus are regulated by CYTOR and translated to the control of downstream effector functions of stimulated T cells. Future studies will further identify the downstream targets of CYTOR that control actin dynamics upon T-cell activation. As additional pathways were identified by our RNA-seq analysis in CYTOR-depleted cells, we visualize that future work will identify novel downstream targets of CYTOR and elucidate their mechanisms of function in regulating HIV gene expression and latency. These may open new ways for developing novel therapeutic tools that will be integrated or substitute current strategies to successfully eliminate the HIV reservoir.

## Materials and methods

### Cells

Jurkat J-Lat 6.3 T cells are immortalized human T lymphocytes that serve as a model for studying HIV latency, as it harbors a transcriptionally silent integrated HIV provirus that encodes for a GFP reporter instead of *Nef*, which reactivated following T cell stimulation. 2D10 cells are also Jurkat-based T cells, carrying a mini—HIV cassette coding for Tat and rev and a 2dGFP reporter gene. Jurkat T cells were maintained in RMPI medium (GIBCO) containing with 10% fetal bovine serum (FBS), 2mg/ml L-glutamine, penicillin-streptomycin, and non-essential amino acids (Sigma, M7145). Cells were cultured at 37°C with 5% CO2. Human Embryonic Kidney HEK293T, this cell line was mainly used for the production of viral-like particles were maintained in DMEM complete medium (GIBCO). Cells were cultured at 37°C with 5% CO2.

### Isolation of primary CD4+ T cells

For the isolation of primary human CD4+ T cells, human Buffy Coats from anonymous healthy donors were obtained from the Soroka Medical Center Hospital Blood Bank. At day 0, PBMCs were isolated over a Ficoll gradient (Millipore). PBMCs were maintained at 2 x10^6^ cells/ml overnight at 37°C. CD4+ T cells were isolated by negative selection with the RosetteSep Human CD4+ T Cell Enrichment Cocktail Stemcell Technologies), resulting in homogenous populations of CD4+ T cells with a purity of 90–95% as assured by flow cytometry. CD4+ T cells were cultured in complete RPMI media containing recombinant human IL2 at 20 U/ml (Roche) to a final concentration 10^6^ cells/ml. Cells were then stimulated with anti-CD3/CD28 dynabeads (Invitrogen) and further cultured for 48 hour. The level of activation was monitored by FACS measuring staining with APC anti human CD25 (Biolegend #302609) and Pacific Blue anti-human CD69 (Biolegend #310919). At day two, stimulated cells were counted, centrifuged for 5 minutes at 1500 rpm, and resuspended in fresh RPMI to a final concentration 0.5x10^6^ cell/ml and IL-2 before transduction with high titter HIV carrying CYTOR shRNA at an MOI of 10. 24 hr later (day three), cells were further transduced with HIV_GKO_ lentivirus at MOI of 10. Transduced cells were cultured in complete RPMI media containing recombinant human IL2 and dynabeads at a ratio of 25 μl human beads per 10 million cells and analyzed by FACS at day five.

### Antibodies

For the IP of P-TEFb, we used the following antibodies: anti-CDK9 (Abcam ab6544) or anti-CYCLIN T1 antibodies (Abcam; ab176702). For ChIP-qPCR for the detection of histone marks activation markers, we used anti-H3K27Ac (ab4729) and anti-H3K4me3 (ab8580). For detecting the different states of the phosphorylation of RNAPII CTD, we used the phosphorylated serine 2 antibody (Ser2P; ab238146) and phosphorylated serine 5 (Ser5P; ab5131). To monitor T cell activation following stimulation, the following antibodies were used: APC anti human CD25 (Biolegend; 302609); Pacific Blue anti human CD69 (Biolegend; 310919).

### Analysis of actin dynamics in response to T cell activation

Actin remodeling in response to T cell receptor (TCR) engagement was monitored by forming circumferential F-actin rings as previously described [61,62]. In brief, stimulatory coverslips were prepared by coating with a 0.01% poly-L-lysine (PLL; Sigma) solution for 10 minutes at room temperature, followed by wet-chamber incubation for 3 hours at 37°C with 7 μg/ml anti-CD3 antibody (50 μl per coverslip, clone HIT3a against CD3E; BD Biosciences) in phosphate-buffered saline (PBS). Stimulatory coverslips were subsequently washed in PBS and stored at 4°C in PBS until use. 5x10^5^ cells per anti-CD3-coated coverslip, respectively) were used to seed coverslips for 4 minutes to allow TCR-mediated actin ring formation. Cells were subsequently fixed in 3% paraformaldehyde for 15 minutes, permeabilized for 2 minutes in 0.1% TritonX-100, and blocked for 30 minutes in 1% Fetal Calf Serum (FCS) in PBS. F-actin was visualized with tetramethyl rhodamine isothiocyanate (TRITC)-conjugated phalloidin (1:1,000, 1 hour, room temperature; Sigma). Samples were mounted on glass slides and analyzed by epifluorescence (Olympus IX81 S1F-3, cellM software) and confocal (spinning-disc PerkinElmer UltraView VoX, Velocity software) microscopes. For quantification of phenotype frequencies, at least 100 transfected cells were counted.

### Generation of pseudotyped lentivirus

Pseudotyped viruses were generated in HEK293T cells as described [19]. Briefly, the plasmid driving the expression of the shRNA transgene was transfected into cells using Lipofectamine 2000 (Invitrogen) together with other lentiviral packaging plasmids coding for Gag, Pol Tat Rev, and the VSV-G envelope. Transfections were done in a 10cm format, and the supernatant containing the virus was harvested 72 hours post-transfection, filtered through a 0.45 μm filter spun at 2000 rpm for 5 min to remove cells debrides and stored at -80°C. 2x10^5^ Jurkat T cells were transduced with the pseudotyped particles for transduction. 16 hours later, the medium containing lentiviral particles was changed. Following transduction, cells were cultured in a medium supplemented with 2 μg/ml of puromycin to eliminate non-transduced cells that did not express shRNA. Upon the death of all the control cells, the medium was changed, and surviving cells were propagated for future experiments.

For transducing CD4 primary cells, we used HIV_GKO_ (a gift from Eric Verdin), which codes for a codon-optimized GFP reporter under the control of the HIV-1 promoter and in the context of expression of all viral proteins and a mKO2 reporter under the control of the constitutive promoter EF1α [48].

### Modulation of CYTOR expression in Jurkat cells and in stimulated primary CD4+T cells

For knockdown (KD) of CYTOR expression, J-Lat 6.3 cells were transduced with lentiviruses that drive the expression of shRNA that specifically targets CYTOR. Cells were next selected on puromycin, and polyclonal stable cells were monitored for CYTOR expression by RT-qPCR. To achieve CYTOR over-expression, cells were transduced with a lentivirus that drives the expression of CYTOR—exons 1, 4 and 5, the most abundantly expressed form in humans. Following antibiotic selection, resistant J-Lat 6.3 T cells were subjected to RT-qPCR to confirm CYTOR over-expression or knockdown. CYTOR RNA levels were normalized to the *gapdh* gene. Although we are aware that GAPDH expression is elevated following TCR stimulation, we did analyze several cellular genes in search of a better marker, but concluded that, e.g., genes for actin polymerization machinery are all affected more strongly by T cell activation than *gapdh* [62]. We therefore used *gapdh* for normalization. To obtain CYTOR knockdown in stimulated primary CD4+ T cells, cells were isolated from health donors (n = 3) and stimulated with anti-CD3/CD28 beads (1:1 ratio of beads to cell number). Cells were cultured on stimulation media (RPMI+IL2), and on day 3 post isolation and stimulation, cells were subjected to transduction with lentivirus expressing shRNA against CYTOR. The following day cells were transduced with HIV_GKO_ and 48 hour later were analyzed by FACS for HIV-GFP and mKO_2_ expression.

### Latency establishment assays

To monitor the effects of CYTOR in promoting HIV latency, we followed the kinetics of entry of stimulated Jurkat 2D10 T cells that express a cassette of the HIV provirus, expressing 2dGFP reporter. Cells where CYTOR is depleted or over-expressed and control cells that express scramble shRNA were activated with P/I and then sorted by FACS to isolate those that express GFP. Cells were then grown, during which their HIV GFP expression was followed by FACS.

### Chromatin Immunoprecipitation (ChIP) analysis

Control cells expressing scramble shRNA or cells where CYTOR expression was depleted (KD) were cross-linked with 1% formaldehyde for 10 minutes and then washed with PBS and reverse cross-linked with glycine (125mM; 5 minutes). Cells were then lysed for 10 minutes on ice in 130μl sonication buffer (20 mM Tris pH-7.8, 2 mM EDTA, 0,5% SDS, 0.5 mM phenylmethylsulfonyl fluoride (PMSF), and 1% protease inhibitor cocktail), and the nuclear pellets were collected. DNA was fragmented by sonication at the following settings: amplitude 20% for 30 cycles at 10 seconds on/10 seconds off. Samples were centrifuged (15 minutes, 14,000 rpm, 4°C). The soluble chromatin fraction (25 μg) was collected and immunoprecipitated (IP) overnight at 4°C on a rotating wheel in IP buffer (0.5% Triton X-100, 2 mM EDTA, 20 mM Tris pH-7.8, 150 mM NaCl and 10% glycerol) with 2.5 μg of one of the indicated antibodies. The next day, the IP material was incubated with 25 μl dynabeads protein G for two hours to ensure the binding of the antibody to the magnetic beads. DNA was eluted with freshly prepared elution solution (1% SDS and 0.1 M NaHCO_3_) and heated at 65°C overnight to reverse-crosslink the samples. Precipitated DNA fragments were then extracted using a ChIP DNA clean and concentrator kit (ZYMO Research), and HIV DNA levels were quantified by qPCR with the primers specifically located on the NFκB region at the HIV-LTR promoter. All signals were normalized relative to input DNA. ChIP assays were also performed with an anti-rabbit or mouse IgG as negative control.

### Chromatin Isolation by RNA Purification (ChIRP)

3x10^6^ cells were cross-linked with freshly made 1% formaldehyde in PBS for 10 minutes at room temperature while shaking. Crosslinking was quenched with 125 μM glycine for 5 minutes at room temperature. Cells were centrifuged at 1200 rpm for 5 minutes at 4°C and washed twice with PBS on ice. The pellet was re-suspended in 300 μl of sonication buffer (50mM Tris 7.0, 10mM EDTA, 1% SDS, DTT, PMSF, protease inhibitors (Roche), and RNase inhibitor (NEB). Cells were then incubated on ice for 10 minutes and sonicated in Bioruptor at high settings of 3 rounds each of 10 cycles 40 seconds ON/40 seconds OFF. Water was changed to ice-cold between the rounds. Sociated samples were centrifuged at max speed for 10 minutes at 4°C, and then chromatin material was kept at -80°C. For IP, chromatin was diluted in twice the volume of hybridization buffer (500 mM, NaCl, 1% SDS, 100 mM Tris pH-8, 10 mM EDTA, 15% Formamide, protease inhibitors (Roche) and RNase inhibitor (NEB).2 μg of biotinylated RNA was added to 0.5 ml diluted chromatin and mixed by end-to-end rotation at 37°C for 4 hours. Streptavidin-magnetic beads were washed three times in sonication buffer, blocked with 500 ng/μl yeast total RNA and 1 mg/ml BSA for 1 hour at room temperature before resuspended in their original volume. 40 μl of beads were added, and the reaction was incubated for 30 minutes at 37°C. Beads were captured by a magnet and washed five times with the wash buffer (2x SSC, 0.5% SDS, supplemented with fresh DTT and PMSF). Beads were resuspended in 3-times of the original volume with the DNA elution buffer (50 mM NaHCO3, 1% SDS, 200 mM NaCl), and DNA was eluted with a cocktail of 100 μg/ml *RNaseA* (Sigma) and 0.1 U/μl RNase H (Epicenter). Chromatin was reverse-cross-by treatment with 0.2 U/μl proteinase K at 65°C for 45 minutes. DNA was then extracted with an equal volume of phenol:chloroform: isoamyl alcohol(Invitrogen) and precipitated with ethanol at -80°C overnight. For probe in-vitro transcription of linear RNA synthesis, 1 μg of RNA was transcribed and biotinylated using AmpliScribe-T7-Flashbiotin-RNA transcription kit (Epicentre) according to the manufacturer’s instructions. Eluted DNA was analyzed by qPCR with primers specific to the HIV promoter.

### RNA Immunoprecipitation (RIP) RT-qPCR

10^7^ Jurkat J-Lat 6.3 cells were washed twice with PBS and resuspended in 800 μl of RNA-IP buffer (0.5% NP-40, 20 mM HEPES pH 7.8, 100 mM KCl, 0.2 mM EDTA supplemented with RNase inhibitor (NEB) and protease inhibitor (Sigma). Cells were cross-linked, and cell lysate was incubated on ice for 10 minutes before isolating nuclei through centrifugation at 2500g for 15 minutes. The supernatant was collected and resuspended in freshly prepared RIP buffer. ChIP material was then sonicated, and the pelleted nuclear membrane and debris were removed by centrifugation at 13,000 rpm for 10 minutes. Isolated ChIP material was incubated with 2.5 μg of indicated antibodies overnight at 4°C. Then, 20 μl of pre-blocked BSA protein A beads were added and incubated for an additional 2 hours at 4°C. 50 μl of cell lysate was collected as input samples. Beads were washed 4 times with washing buffer (0.5% NP-40, 20 mM HEPES pH 7.8, 100 mM KCl, 0.2 mM EDTA supplemented with RNase inhibitor (NEB) and protease inhibitor (Sigma) to remove unbound material. The pellet was resuspended in 100 μl of the lysis buffer and extracted using a TRIZOL reagent (Sigma). RNA was reverse transcribed using qPCRBIO kit (PCPbiosystems), and qPCR was performed using indicated primers against CYTOR or 7SK ncRNA. The amplification of 7SL RNA served as a control RNA that is not associated with P-TEFb. Input RNA was extracted and reverse-transcribed the same way. Dilutions of input were used for standard curve and calculations.

### RNA pull-down and western blotting

10^7^ Jurkat cells were washed twice with PBS and resuspended in 800 μl of RNA-pull-down buffer (0.5% NP-40, 20 mM HEPES pH 7.8, 100 mM KCl, 0.2 mM EDTA supplemented with RNase inhibitor (NEB) and protease inhibitor (Sigma). Lysates were incubated with an in-vitro transcribed biotinylated CYTOR anti-sense probe (synthesized by IDT), and reactions were pulled down with streptavidin beads. Beads were resuspended in 3 times of their original volume of DNase buffer (100 mM NaCl and 0.1% NP-40), and protein was eluted with a cocktail of 100 μg/ml *RNaseA* (Sigma) and 0.1 U/μl *RNaseH* (Epicenter) and 100 U/ml DNase I (Invitrogen) at 37°C for 30 minutes. Eluted proteins were subjected to western blotting with indicated antibodies. Non-specific IgG served as control. Biotinylated scrambled RNA was used as a control for RNA-IP. 7SK RNA confirmed association with P-TEFb. Input is 5% of the total cell lysate [63].

### Nuclear and cytoplasmic biochemical fractionation

The cytosolic extracts were prepared by resuspending 3x10^6^ cells in 500μl of Buffer A (10 mM KCl, 10 mM MgCl_2_, 10 mM HEPES, 1 mM EDTA, 1 mM DTT, 0.1% PMSF, and EDTA-free complete protease inhibitor cocktail (Roche) with 0.5% NP-40 for 10 minutes on ice. The nuclei were spun down at 5,000 *g* for 5 minutes, and the supernatant was saved as the cytosolic extract (CE). The nuclei were washed once with 200 μl of Buffer A with 0.5% NP-40 and re-pelleted. The nuclei were resuspended in 100 μl of Buffer B (450 mM NaCl, 1.5 mM MgCl_2_, 20 mM HEPES, 0.5 mM EDTA, 1 mM DTT, 0.1% PMSF, and EDTA-free complete protease inhibitor cocktail (Roche) and incubated on ice for 60 minutes. The lysates were clarified by centrifugation at 20,000*g* for 10 minutes to prepare nuclear extract (NE). RNA from nuclear or cytoplasmic was extracted with Trizol, and RNA was reverse transcribed using a qPCRBIO kit (PCPbiosystems), and qPCR was performed using indicated primers against CYTOR or 7SK ncRNA. CYTOR and 7SK RNA levels were normalized to 7SL RNA in each of the fractions and conditions.

### RNA purification for RNA sequencing

For identifying ncRNAs that are differentially expressed upon T cell stimulation in cells that carry active HIV (GFP+) *versus* latent HIV (GFP-), J-Lat 6.3 cells were stimulated with P/I and sorted based on their GFP expression (n = 4). For analysis of transcriptome upon CYTOR depletion cells, primary CD4+ T cells were stimulated with CD3/CD28 beads and then subjected to CYTOR KD by transducing cells with lentivirus that drive the expression of shRNA that target CYTOR (n = 3). CYTOR overexpression was obtained by transducing stimulated cells with lentivirus that drive CYTOR expression. RNA was purified utilizing the RNeasy Mini kit (QIAGEN) according to the manufacturer’s instructions. The integrity of the isolated RNA was tested using the Agilent High Sensitivity RNA Kit and Tapestation 4200 at the Genome Technology Center at the Faculty of Medicine Bar-Ilan University. Total RNA was used for mRNA enrichment by using the NEBNext mRNA polyA Isolation Module (NEB; E7490L), and libraries for Illumina sequencing were performed using the NEBNext Ultra II RNA kit (NEB; E7770L). Quantification of the library was performed using dsDNA HS Assay Kit and Qubit 2.0 (Molecular Probes, Life Technologies), and qualification was done using the Agilent D1000 Tapestation Kit and Tapestation 4200. 150 nM of each library was pooled together and was diluted to 4nM according to NextSeq manufacturer’s instructions. 1.6 pM was loaded onto the Flow Cell with 1% PhiX library control. Libraries were sequenced on an Illumina NextSeq 500 instrument, 75 cycles of single-read sequencing.

For analysis: Quality Control was conducted by evaluating the quality of the FASTQ files using ’Fastqc’ (v0.12.1). Subsequently, the samples underwent quality trimming via the ’Fastp’ software (v0.23.3). To identify potential contaminations, ’fastq-screen’ software was applied. Read alignment involved aligning the reads to the Human GRCh38 genome (Ensembl release 110) using ’STAR’ software (v2.7.10b). Read assignments to coding regions were determined using the ’SubRead’ package (’FeatureCounts’ v2.0.6). Finally, BAM files were sorted using ‘samtools’. Differential expression analysis was conducted in DAVID. PCA was performed to evaluate data dispersion, revealing a batch effect among samples collected on different dates. A correction was implemented in the DESeq2 model to minimize this batch effect. Finally, the indicated groups were compared (without including the control in the model), identifying 90 DEGs. The selected cutoff values were an adjusted p-value <0.05 and a fold-change > 2. For enrichment analysis, the DAVID program was employed to identify enriched pathways and terms associated with the selected genes [64,65]. These quality control and data analysis steps ensure the reliability and accuracy of the RNA-seq analysis.

### Primers used for qPCR analysis

Primers on the HIV promoter:

NFκB forward: 5’ - AGGTTTGACAGCCGCCTA -3’

NFκB Reverse: 5’ - AGAGACCCAGTACAGGCAAAA -3’

gapdh Forward: 5’ - AGCCACATCGCTCAGACAC -3’

gapdh Reverse: 5’ - GCCCAAACGACCAAATCC -3’

Primers for CYTOR:

Forward: 5’- AACTTGCCAGCCTCCATC;

Reverse: 5’- GAGCTTCCTGTTTCATCTCCC

Primers for 7SK:

Forward; 5‘- GAGGGCGATCTGGCTGCGACAT

Reverse: 5‘- ACATGGAGCGGTGAGGGAGGAA

### Statistical measurements

Statistical evaluation was performed with GraphPad Prism 7 using two-way ANOVA with no correction for multiple comparisons. Number of independent data points refers to biological replicates. Each data point, as mentioned in the figure legends, represents the mean of 3–4 independent experiments with the errors calculated based on mean ± SD. Differences were considered statistically significant and denoted as ***p≤0.05; n.s., not significant.

## Supporting information

S1 Fig Staining for activation markers of stimulated CD4+ primary cells infected with active or latent HIV. (TIF)

S2 Fig RNA levels of CYTOR do not change upon HIV infection. (TIF)

S3 Fig Promoter occupancy of CYTOR on P-TEFb target genes. (TIF)

S4 Fig Staining for activation marker of stimulated CD4+ primary cells upon modulation of CYTOR expression. (TIF)

S5 Fig Effect of CYTOR overexpression on actin dynamics in CD4+ T cells. (TIF)

S1 Table List of genes from RNA-Seq of Jurkat T cells that carry active or latent HIV. (XLSM)

S2 Table List of genes from RNA Seq of CD4+ primary cells upon CYTOR manipulation of expression. (XLSM)
