# Supplementary figures and images for: Direct and indirect effects of CYTOR lncRNA regulate HIV gene expression
Source: PLoS Pathog. 2024 Apr 25;20(4):e1012172. doi: 10.1371/journal.ppat.1012172 (PMC11075828; doi:10.1371/journal.ppat.1012172)

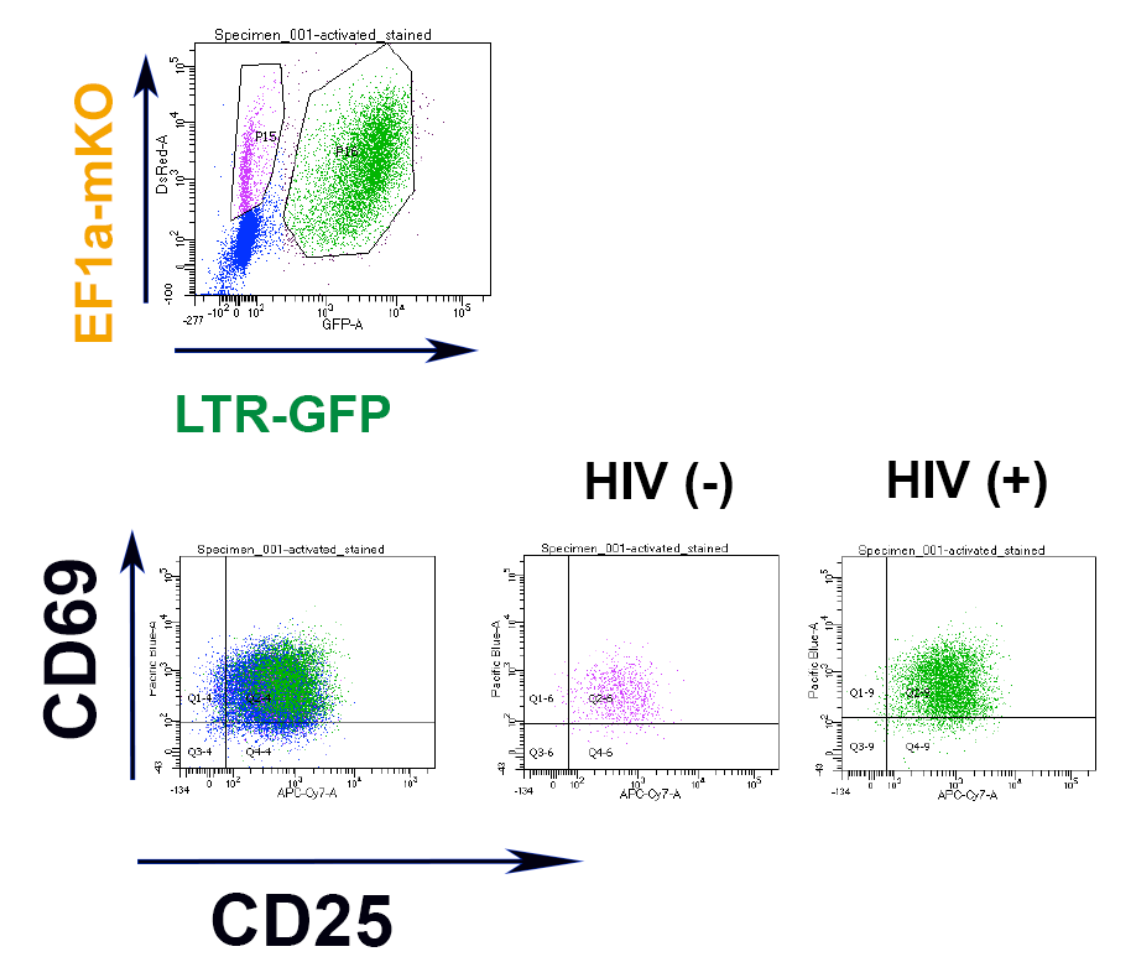

Supplement: S1 Fig — (TIF) [file ppat.1012172.s001.tif]

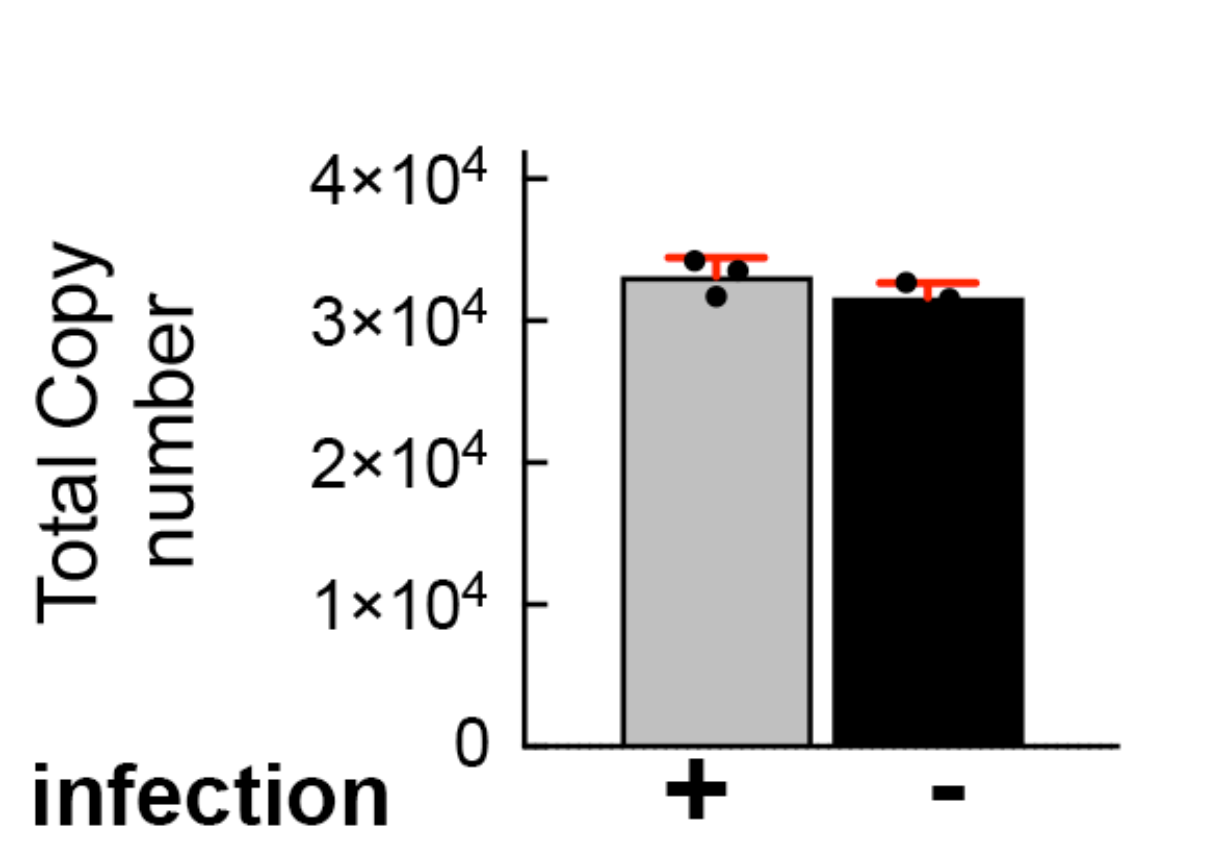

Supplement: S2 Fig — (TIF) [file ppat.1012172.s002.tif]

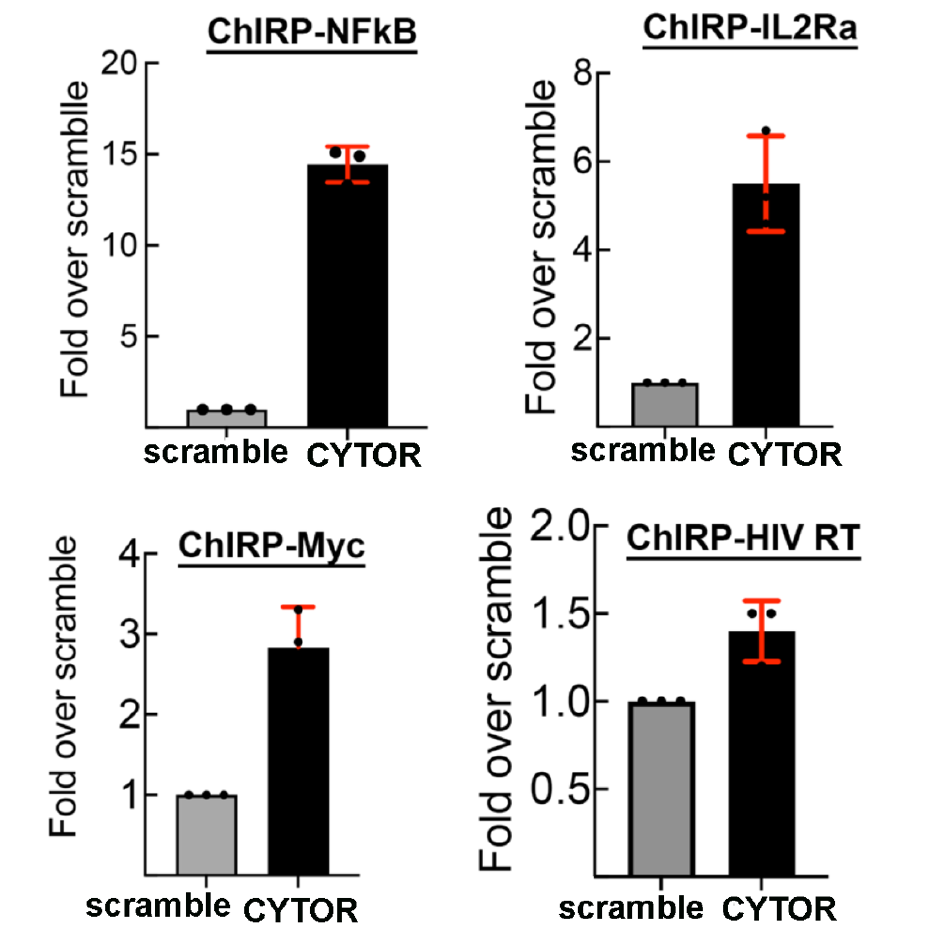

Supplement: S3 Fig — (TIF) [file ppat.1012172.s003.tif]

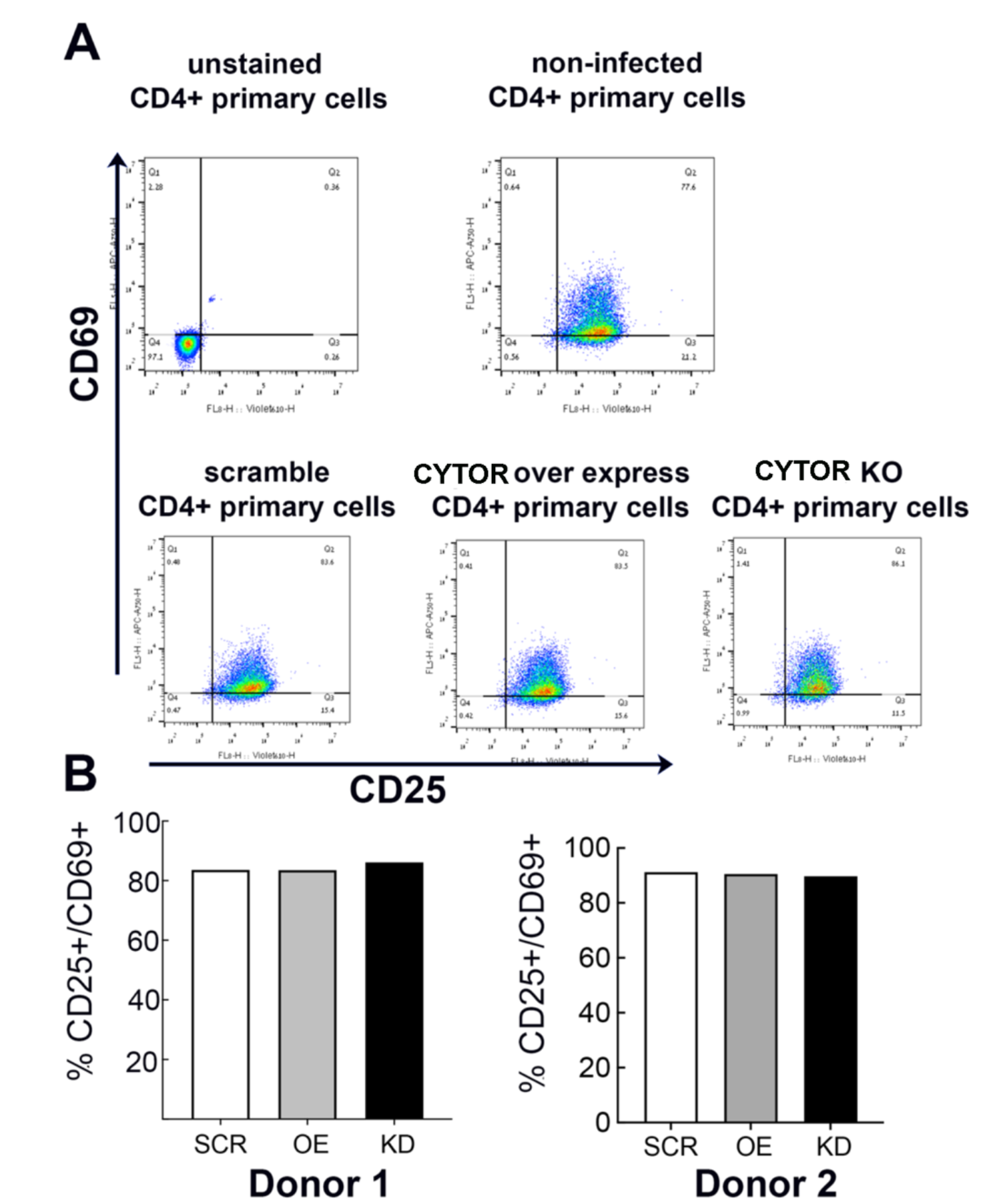

Supplement: S4 Fig — (TIF) [file ppat.1012172.s004.tif]

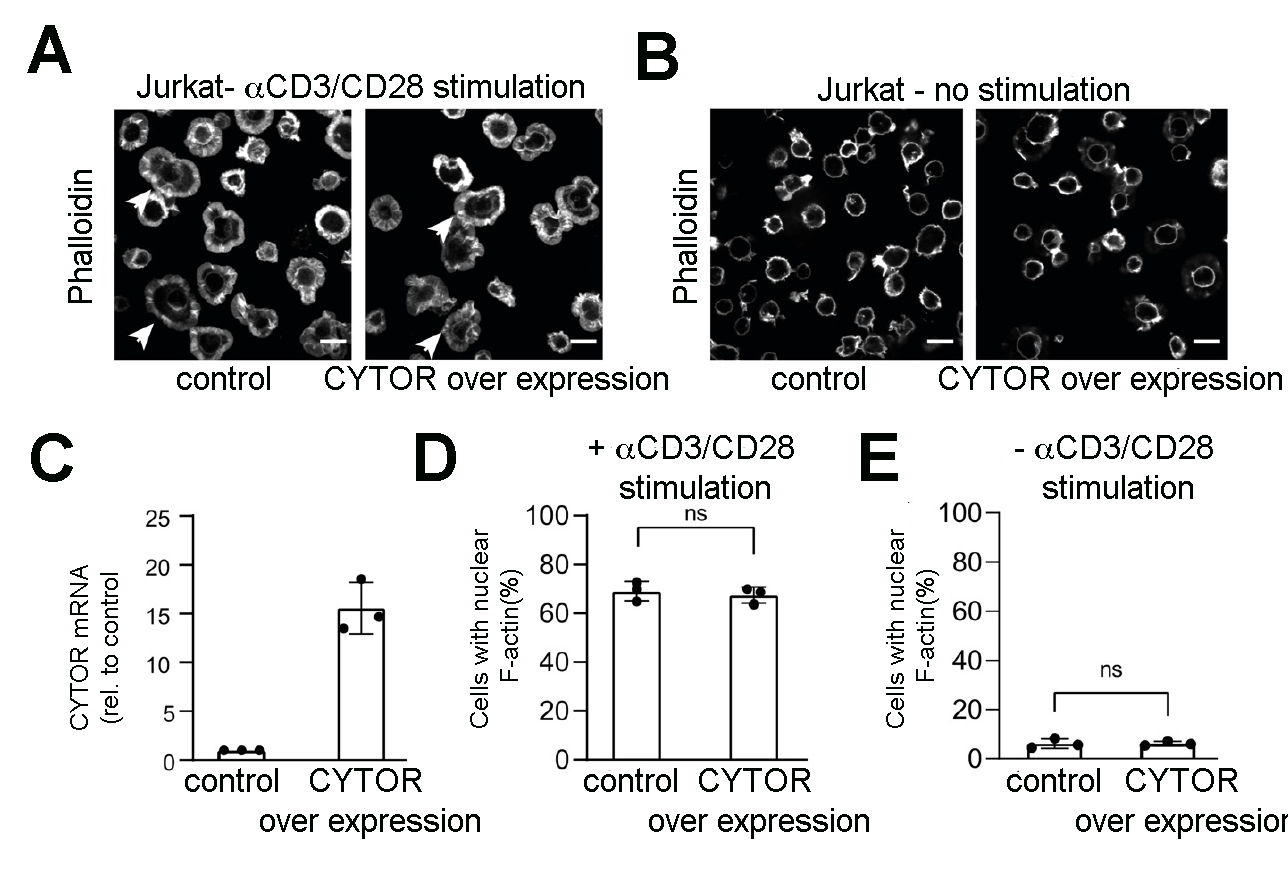

Supplement: S5 Fig — (TIF) [file ppat.1012172.s005.tif]
